# Chiral phthalimides against penicillin-binding protein 2a of methicillin-resistant *Staphylococcus aureus*: molecular docking and *in vitro* analysis

**DOI:** 10.3389/fphar.2024.1293458

**Published:** 2024-02-28

**Authors:** Aamina Azam Khan, Momin Khan, Sher Wali Khan, Nayyer Siddique, Rimsha Abid, Shandana Zulfiqar, Sidra Rahman, Muhammad Ali

**Affiliations:** ^1^ Institute of Pathology and Diagnostic Medicine, Khyber Medical University (KMU), Peshawar, Pakistan; ^2^ Department of Microbiology, Khyber Medical University (KMU), Peshawar, Pakistan; ^3^ Department of Chemistry, Rawalpindi Women University, Rawalpindi, Pakistan; ^4^ Institute of Basic Medical Science, Khyber Medical University (KMU), Peshawar, Pakistan; ^5^ Department of Biotechnology, Quaid-i-Azam University Islamabad, Isalmabad, Pakistan

**Keywords:** methicillin-resistant gene, penicillin-binding protein2a, methicillin-resistant *Staphylococcus aureus*, chiral phthalimides, molecular docking, cytotoxicity

## Abstract

*Staphylococcus aureus* (*S. aureus*) is a commensal bacterium and an opportunistic pathogen causing a wide variety of infections ranging from localized skin and soft tissue infections to life-threatening severe bacteremia, osteomyelitis, endocarditis, atopic dermatitis, prosthetic joint infection, staphylococcal food poisoning, medical device-related infections, and pneumonia. It is attributed to an acquired resistant gene, *mecA*, encoding penicillin-binding protein 2a (PBP2a). PBP2a is an essential protein responsible for the resistivity of methicillin-resistant *S. aureus* (MRSA) to various beta-lactam antibiotics. The antimicrobial treatment alternatives for MRSA are increasingly limited. Therefore, developing alternative therapeutic options for its treatment is the need of the day. Phthalimides and their N-substituted derivatives are of biological importance as they possess extensive biological and pharmaceutical properties and can serve as an excellent therapeutic option for MRSA. This study uses three chiral phthalimides (FIA, FIB, and FIC) to check their *in silico* and *in vitro* inhibitory effects. Molecular docking of these chiral phthalimides against PBP2a of MRSA was performed initially. After promising results, these novel compounds were screened through the agar-well diffusion method and micro-broth dilution assay to investigate their *in vitro* inhibitory activities with FIB being the strongest anti-staphylococcal agent yielding a 21 mm zone of inhibition and a minimum inhibitory concentration (MIC) of 0.022 ug, respectively. The zones of inhibition obtained through the *in vitro* activity showed that these chiral phthalimides possess substantial anti-MRSA activities and have the potential to be considered as alternative chemotherapeutics to treat the infections caused by MRSA after the confirmation of their cytotoxic and pharmacokinetic studies.

## Introduction

Genus *Staphylococcus* contains gram-positive, bacitracin-resistant, catalase-positive, non-spore-forming coccus bacteria (Cheung et al., 2021). It is a coagulase-positive bacterium, which is the common colonizer of the human body. It is a highly virulent pathogen of “Risk Group Level 2” because of its ability to invade, persist, replicate, and infect human tissues (Wang et al., 2016). It is an opportunistic pathogen responsible for several human infections, from localized skin and soft tissue infections to life-threatening severe bacteremia (Matuszewska et al., 2020), endocarditis, osteomyelitis, atopic dermatitis, staphylococcal food poisoning, staphylococcal toxic shock syndrome (TSS) (Turner et al., 2019), prosthetic joint infections, medical device-related infections, and pneumonia (Senobar Tahaei et al., 2021). Due to its toxin-producing abilities (i.e., staphylococcal enterotoxin), it is a significant cause of staphylococcal foodborne disease (Fri et al., 2020). *S. aureus* can also cause infections in livestock, like mastitis (in ruminants) and bumblefoot (in poultry), as well as diseases in domestic animals (dogs and cats), resulting in substantial morbidity rates in animals and a significant economic loss to the farmers.

Methicillin-resistant *S. aureus* (MRSA) was first isolated in 1961 in a hospital in the UK (Ullah et al., 2016). MRSA is resistant to almost all β-lactams (Craft et al., 2019), including penicillin, trimethoprim, erythromycin, clindamycin, and tetracyclines (Turner et al., 2019), hence minimizing the safe and effective treatment options for MRSA. These strains also resist other antibiotic groups, e.g., fluoroquinolones, macrolides, and aminoglycosides, and are, therefore, known as “Superbug” (Senobar Tahaei et al., 2021). The success of this pathogen is related to the presence of its extensive virulence factors such as enzymes (hyaluronic acid, lipases, proteases, coagulase, and staphylokinase), toxins (hemolysins and leukocidins), antigens, and evasive immune factors (protein A and capsule) coupled with its remarkable ability to quickly acquire resistance to different antibiotics used in clinical practice over the years (Fri et al., 2020).

Methicillin resistance is associated with the modifications in penicillin-binding proteins (PBP2a/2c/2ʹ), due to the presence of *mecA*, *mecB*, or *mecC* genes in *S. aureus*. In addition to PBP1-4, MRSA strains encode a high-molecular-weight fifth penicillin-binding protein, called penicillin-binding protein 2a (PBP2a), responsible for their resistance (Fergestad et al., 2020). PBP2a is encoded by the *mecA* gene, which is present inside a mobile genetic element known as the *Staphylococcal* cassette chromosome mec (SCC*mec*) (Miragaia, 2018; Craft et al., 2019). PBP2a enables the synthesis of the bacterial cell wall in the presence of antibiotics, as it has a low affinity for all beta-lactam antibiotics, thus allowing cross-linking to proceed (Craft et al., 2019; Fergestad et al., 2020). Like other PBPs, the active site of PBP2a contains the active site serine (Ser403) located at the N-terminus. Still, it lies deep in the pocket and is inaccessible to beta-lactam antibiotics. This closed active site indicates the resistance offered by PBP2a (Bien et al., 2011). Moreover, some additional genes essential for methicillin resistance, such as factors necessary for the expression of methicillin resistance (*fem*) and auxiliary factors (*aux*), have also been identified (Bien et al., 2011).

The common antibiotics used against MRSA currently include daptomycin, gentamicin, vancomycin, linezolid, TMP–SMZ (trimethoprim/sulfamethoxazole), tigecycline, clindamycin, and amikacin, but these antibiotics are losing their efficacy (Okwu et al., 2019). In addition, teicoplanin, daptomycin, and linezolid are expensive (Okwu et al., 2019). As the therapeutic options available at present for the treatment of MRSA infections are limited, there is an urgent need to develop novel drugs effective for treating MRSA infections (Okwu et al., 2019). Cyclic imides and their N-substituted derivatives [-CO-N(R)-CO-] are an important class of organic compounds containing bis-amide linkages. Their neutral structures and hydrophobicity allow them to cross biological membranes easily. The nitrogen and oxygen atoms as donor sites can co-ordinate these molecules with the biological system causing some pharmacological effects (Hassoun et al., 2017). Among the imide-ring-containing heterocyclic compounds, phthalimides and their N-substituted derivatives possess extensive biological and pharmaceutical properties. They have also been used as tumor necrosis factor-alpha (TNF-alpha) inhibitors, which has a crucial role in several physiological immune systems (Kushwaha and Kaushik, 2016). These molecules are reported to possess important biological properties including antifungal, antibacterial, antiviral, anticancer, analgesic, antidepressant, apoptosis induction, anti-inflammatory, androgen receptor antagonist, anxiolytic, anticonvulsant, and muscle relaxant activities (Jafari et al., 2017). The antibacterial properties of different phthalimide derivatives of N-alkyl, N-alkyloxy, N-acyl, butyl, and N-hydroxyl have been reported against both Gram-positive (*Streptococcus pneumoniae*, *Micrococcus luteus*, and *Listeria monocytogenes)* and Gram-negative (*Pseudomonas aeruginosa*, *Salmonella typhimurium*, and *Escherichia coli)* bacterial strains which have shown promising results because of their smaller size and high potency in disease control management (Othman et al., 2019). Similarly, the derivatives of chiral phthalimides with N-alkyloxy and N-alkyl substitution in combination with maleimide have shown antifungal activity against fungi (*Candida albicans* and *Aspergillus fumigatus)* (Imran et al., 2019). One of the reasons for inflammatory diseases is the denaturation of the protein present in tissues that leads to the development of autoantigens. Phthalimide derivatives with the combination of N-aryl or aryl amines in the presence of an L-proline catalyst can prevent this denaturation process and, hence, inhibit the development of inflammatory diseases (Perveen and Orfali, 2018). So, these compounds can serve as an excellent therapeutic approach for treating MRSA infections; therefore, our study used three different chiral phthalimides to check their *in silico* and *in vitro* inhibitory effects. Regardless of the enormous investments and the time consumed in discovering new drugs, its success rate through clinical trials is only 13%, with a relatively high failure rate. These failures have been reported at a later stage in the majority of cases (40%–60%) because of the lack of optimum pharmacokinetic properties (absorption, distribution, metabolism, excretion, and toxicity) of the drugs (Gurung et al., 2021). So, recently, a decrease in the market has been noted in the number of new drugs because they failed in different phases of the clinical trials. Therefore, it is essential to cope with the limitations in the conventional drug discovery methods with a cost-effective, efficient, and broad-spectrum computational drug development process. Such computational techniques will aid the drug discovery and development process by minimizing the costs and final stage failure chances when used by various pharmaceutical companies and research groups in the preliminary studies (Batool et al., 2019; Gurung et al., 2021).

Molecular docking is a structure-based drug-designing approach (SBDD). It was first developed for predicting the binding action of known active molecules and virtual screening of sizeable digital compound libraries to minimize the cost and speed up drug discovery processes (Torres et al., 2019). It has been widely used as an inexpensive and fast technique in industrial and academic settings. The objective of molecular docking is to get an optimized conformation for both the ligand and the protein and the relative orientation between the ligand and the protein in such a manner that the free energy of the overall system is minimized (Torres et al., 2019). This drug-designing approach is effectively fast and specific for the identification and opsonization of lead molecules, which has helped to understand the molecular level of a disease (Gurung et al., 2021). In the current study, the chiral phthalimides (FIA, FIB, and FIC) used were docked against the PBP2a protein of MRSA before their *in vitro* antibacterial activity. The reason for selecting these chiral phthalimides was their good antibacterial activity against several Gram-positive and Gram-negative bacteria present in the literature.

## Materials and methods

### Molecular docking analysis

#### Retrieving the 3D structure of the ligands

All three chiral phthalimides’ chemical structures were retrieved from PubChem (https://pubchem.ncbi.nlm.nih.gov/). Their PubChem IDs are FIA (ID = 334207), FIB (ID = 688554), and FIC (ID = 1714209).

### Structure retrieval of PBP2a

The sequence of the protein PBP2a was retrieved from the NCBI (Accession No. ALJ 10988.1). This sequence was saved in the FASTA format, which gives information regarding the total number of amino acids present in the sequence and other related information.

### Homology modeling and structure evaluation

Since no information was present in the Protein Data Bank (PDB) (www.rcsb.org), the protein was modeled using SWISS-MODEL to deduce the structure of the protein PBP2a. SWISS-MODEL is consistently ranked among the top modeling servers for several crucial modeling aspects, and it deduces the protein structure on the basis of the already available protein structures (homology modeling). The structure obtained through modeling was then evaluated using PROCHECK (Laskowski et al., 1996) for checking the accuracy of stereochemical parameters (e.g., bond length, G factor, conformational angles, and bond angles). The relationship of the sequence with the 3D structure was verified utilizing VERIFY3D (Eisenberg et al., 1997). The ERRAT scheme plot was also performed to verify protein structure validity on the basis of crystallography, and structure structures generally produce values around 95% or higher, considered suitable high-resolution structures. The Ramachandran plot was generated to predict the structural stereochemical property (i.e., structures that score more than 85% will be considered valid). The assessment and validity of the structure was also carried out again once the minimization of the structure was carried out. This step-by-step procedure validated the protein structure of PBP2a, and a high-quality model was generated at the end.

### Energy minimization

Once the structure modeling is performed, a crucial step, i.e., energy minimization, must be performed, removing all the torsions within a molecule and bringing it to the lowest possible energy state to ensure a good structure. To accomplish energy minimization of the structure, ModRefiner adjusted the root mean square deviation (RMSD) value for the refined structure by changing the bond angles of the unrefined structure of PBP2a.

#### Binding pocket determination

For determining the active sites of the PBP2a protein of MRSA, CASTp (Binkowski et al., 2003; Raffo et al., 2022) and ProteinsPlus (Schöning-Stierand et al., 2020) (https://proteins.plus/) were used by uploading the file of the protein structure in the .pdb format. The residues in the pocket were then noted.

### Molecular docking

For docking the chiral phthalimides with the active sites of PBP2a, PatchDock (Duhovny et al., 2002) was used. At one time, .pdb formats of the targeted protein and only one ligand were uploaded for docking. The BIOVIA Discovery Studio (DS Visualizer) 2021 Client was finally used for analyzing the results of the docked molecules obtained.

### *In vitro* study analysis

#### Sample collection and processing

It is an experimental study for which ten MRSA samples were collected from the microbiology laboratory of Hayatabad Medical Complex. The samples were collected from different sites such as wound, pus, and high vaginal swabs. Those samples were further processed in the microbiology laboratory of IPDM, KMU, according to the laboratory-optimized protocol. The confirmed strains of MRSA were stored in the KMU repository with strain numbers from AAK001IPDM to AAK010IPDM.

#### Inoculation on different growth media and the identification of *S. aureus*

All the samples obtained were inoculated on MSA (Mannitol salt agar) and blood agar plates. The identification of *S. aureus* was made based on morphological features and biochemical tests such as Gram staining, catalase, and coagulase tests.

#### Antibiotic susceptibility testing

The standardized method, i.e., the Kirby–Bauer disc agar diffusion method, was performed using different antibiotic discs on Mueller–Hinton agar (MHA) to differentiate MRSA from MSSA samples. The Clinical and Laboratory Standard Institution Guidelines (CLSI) 2022 established the susceptibility pattern. Different antibiotic discs, including cefoxitin (FOX- 30 µg), ciprofloxacin (CIP- 5 µg), penicillin (P- 10 ug), vancomycin (V- 30 µg), gentamicin (CN- 10 µg), erythromycin (E− 15 ug), amikacin (AK- 30 ug), cefepime (CPM- 30 ug), clindamycin (DA- 2 µg), and linezolid (LZD- 30 µg), were used.

#### Antibacterial activity of chiral phthalimides

The antimicrobial activity of the chiral phthalimides was checked against *S. aureus* (MRSA) through the agar-well diffusion method using Muller–Hinton agar, which is commonly used for checking the antibacterial activities of novel compounds. Following the standard protocol, 5000 ug (5 mg) of each of the compounds were dissolved in 1000 uL (1 mL) of DMSO, and a final volume of approximately −250–300 uL was added to the wells (−4 mm diameter) made on agar plates. 100% DMSO was used as a control for this assay.

#### Chiral phthalimides used in the current study

i) Compound 1 (FIA): [2-(1, 3-Dioxoisoindolin-2-yl)-3-phenylpropanoic acid]
ii) Compound 2 (FIB): [2-(1, 3-Dioxoisoindolin-2-yl)-4-methylpentanoic acid]
iii) Compound 3 (FIC): [2-(1, 3-Dioxoisoindolin-2-yl)-4-(methylthio) butanoic acid]

#### Determining the minimum inhibitory concentration

The 96-well plate assay was used to determine the minimum inhibitory concentration (MIC) of the compounds. It is a well-known worldwide method for determining the minimum inhibitory concentrations of the test compounds, that is, the minimum concentration of test compounds required to inhibit the visible growth of bacteria. This technique used a sterilized 96-well plate with proper positive (McFarland standard) and negative (nutrient broth) controls. Each well has a total capacity of holding 200 ul of the solution.

#### Determining the minimum bactericidal concentration

For identifying the minimum bactericidal concentration (MBC), a visual plate assay was performed on the MHA and blood agar plates.

## Results

### Molecular docking analysis

#### 3D structures of the ligands obtained

The structures of ligands retrieved via PubChem are given in Table 1.

**TABLE 1:** Ligands and their 3D structures.

Name of the ligand	3D picture
Compound 1: FIA [2-(1, 3-dioxoisoindolin-2-yl)-3-phenylpropanoic acid]	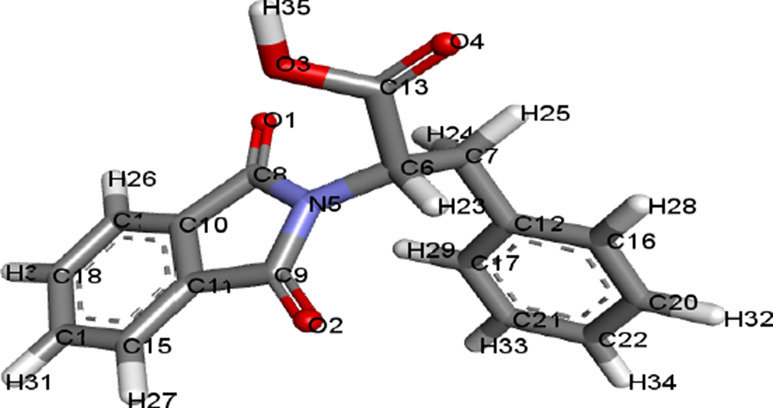
Compound 2: FIB [2-(1, 3-dioxoisoindolin-2-yl)-4-methylpentanoic acid]	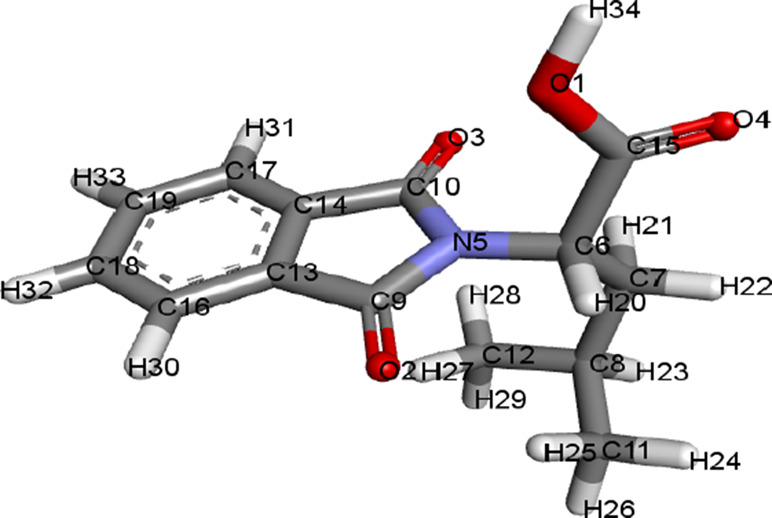
Compound 3: FIC [2-(1, 3-dioxoisoindolin-2-yl)-4-(methylthio) butanoic acid]	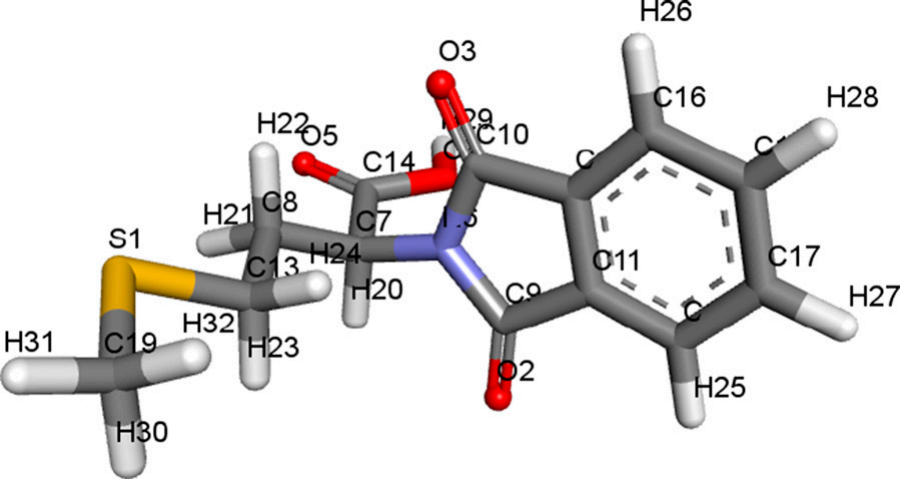

#### Homology modeling and structure evaluation

**FIGURE 1:**
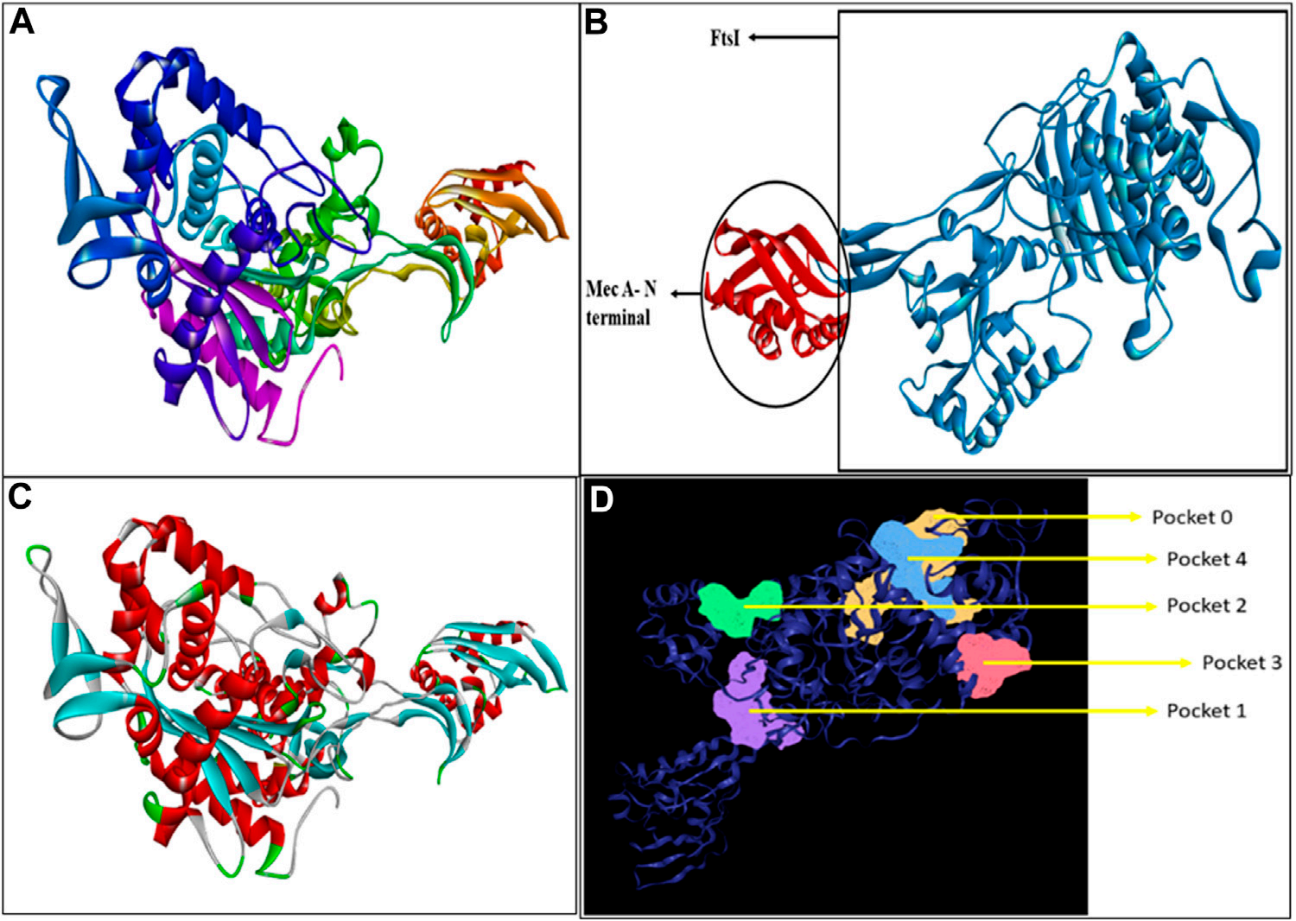
**(A)** Modeled 3D structure of chain A of PBP2a. **(B)** Labeled 3D structure of PBP2a showing two different regions; red color indicates the N-terminal of mecA (amino acids 25–140), and blue color indicates the conserved protein domain FtsI (amino acids 136–667). **(C)** Energy-minimized refined model of PBP2a. **(D)** Structure of PBP2a with its five active pockets.

Penicillin-binding protein 2a contains a single amino acid chain (chain A) totaling 668 amino acids. The 3D structure of PBP2a was obtained through SWISS-MODEL Figure 1A. Figure 1B shows the labeled 3D structure of PBP2a in which the red color indicates the N-terminal of mecA (comprising amino acids 25–140), whereas the blue color indicates the conserved protein domain FtsI (containing amino acids 136–667).

**FIGURE 2:**
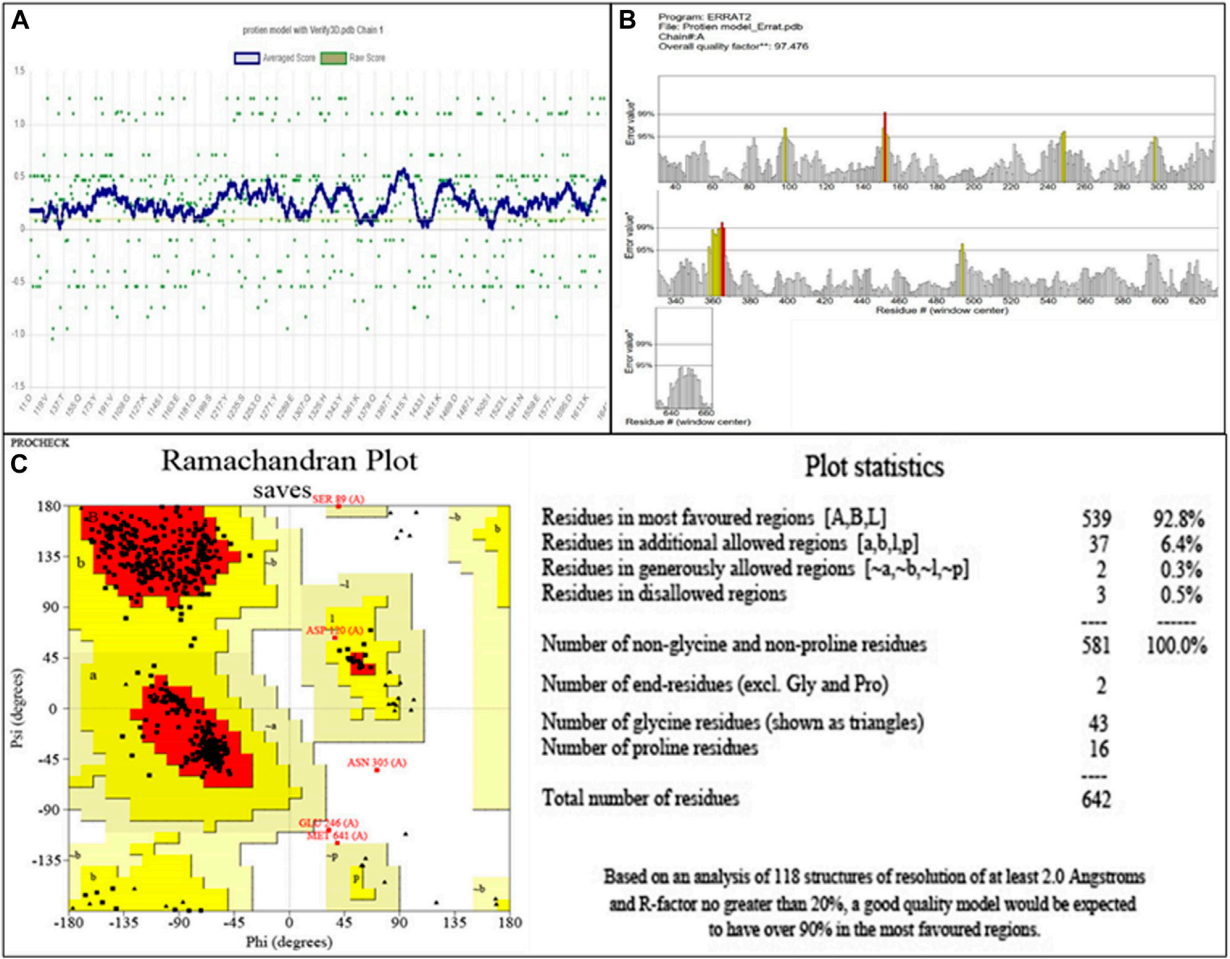
**(A)** Validation of 2D–3D construction via VERIFY3D. Blue color indicates the average residue score, while green dots represent the raw residue score. **(B)** The ERRAT scheme plot shows the overall quality of the protein model with 97.47%, which is higher than 95%, indicating the good high-resolution structure. *On the error axis, two lines are drawn to represent the confidence with which regions exceeding the error value can be rejected. **, expressed as the proportion of proteins for which the estimated error value falls below the 95% rejection threshold. Good high-resolution structures typically produce values of at least 95 percent. For lower resolutions (2.5–3A), the average quality factor is approximately 91%. **(C)** The Ramachandran plot showing amino acid residues in different regions for the evaluation of PBP2a. Red color indicates the most favored region that is 95.0%. Dark yellow color indicates the additional allowed region that is 4.1%, while light yellow and white colors indicate the generously allowed region and disallowed region that are 0.3% and 0.5%, respectively.

The modeled protein structure was further verified through VERIFY3D, as shown in Figure 2A. The blue line indicates the average score of the actual 3D protein model. In contrast, the green dots mark the raw scores for our protein model generated. The VERIFY3D score showed that 92.37% of the residues had scored >=0.1 and are compatible with the average score, so our protein model is acceptable. This further validates the 2D–3D construction (compatibility of the 2D sequence to the 3D structure). The general criteria for the structures to be considered as PASS in VERIFY3D is that at least 80% of the amino acids should score >=0.1 in the 3D/1D profile. Since our structure fulfilled this criterion, it was considered as PASS. The ERRAT scheme plot further validated the structure and showed a good high-resolution structure with a value score of 97.47%, more than 95%, as shown in Figure 2B.

The evaluation of the modeled structure through PROCHECK produced a Ramachandran plot, as shown in Figure 2C. The results from the Ramachandran plot indicated that 92.8% of the residues reside in the most favored region (red), hence indicating the suitable stereochemical parameters for the structure of PBP2a, whereas 6.4% of the residues reside in the additional allowed region (dark yellow), 0.3% residues reside in the generously allowed region (light yellow), and 0.5% residues reside in the disallowed region (white).

#### Energy minimization

Energy minimization through ModRefiner adjusted the RMSD (root mean square deviation) value for the refined structure to 0.440, as shown in Figure 1C, which was further used for docking.

#### Binding pocket determination

A total of five active pockets in PBP2a were determined using ProteinsPlus, as shown in Figure 1D by different colors. The properties of those pockets are summarized in Table 2 (Supplementary Table S1 for the amino acids in the binding pockets is attached in Supplementary Material).

**TABLE 2:** Properties of the active pocket of PBP2a.

S. no.	Active pocket	Volume A^3^	Surface A^2^	Drug score
1	Pocket 3	441.26	791.51	0.84
2	Pocket 0	1412.11	1647.33	0.8
3	Pocket 4	365.22	373.88	0.77
4	Pocket 1	932.15	1145.63	0.76
5	Pocket 2	553.97	727.26	0.71

#### Molecular docking

**FIGURE 3:**
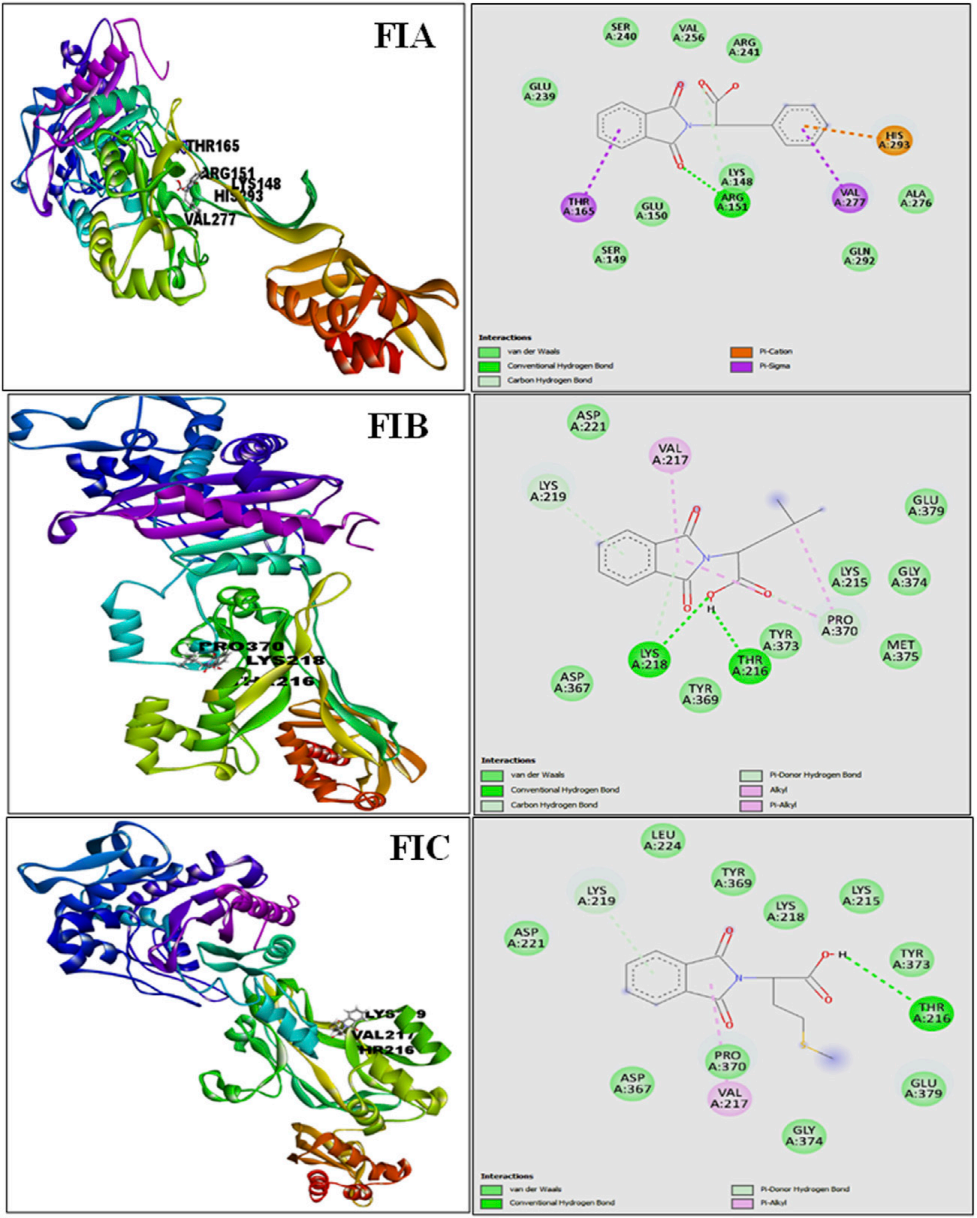
Complete 3D structure of FIA, FIB, and FIC docked with PBP2a (left) and their respective 2D diagram showing hydrogen bonding and the specific amino acids involved (right). It is shown that compound 1 (FIA) formed one hydrogen bond (ARG-151), compound 2 (FIB) formed two hydrogen bonds (THR 216, LYS 218), and compound 3 (FIC) also formed one hydrogen bond (THR 216) with the PBP2a.

The results from PatchDock showed that compound 1 (FIA) formed one hydrogen bond (ARG-151), compound 2 (FIB) formed two hydrogen bonds (THR 216, LYS 218), and compound 3 (FIC) also formed one hydrogen bond (THR 216) with the PBP2a (Figure 3). The basis of the selection of the compounds was the number of hydrogen bonds it formed with the PBP2a. The more the number of hydrogen bonds, the strongest is the compound. In addition, those compounds which interacted with the PBP2a protein through a covalent bond were rejected because of the chances of their off-site toxicity in the host. Both the 2D and 3D confirmations of the ligand and protein along with the hydrogen bonds involved are shown. Table 3 summarizes docking parameters for the selected chiral phthalimides with PBP2a.

**TABLE 3:** Docking parameters.

Ligand name	Score	Area	ACE	Number of hydrogen bonds	Number of covalent bonds
Compound 1 (FIA)	3594	451.70	−101.27	1	0
Compound 2 (FIB)	3468	372.90	−110.25	2	0
Compound 3 (FIC)	3592	378.60	−132.78	1	0

### *In vitro* study analysis

#### Inoculation on different growth media

**FIGURE 4:**
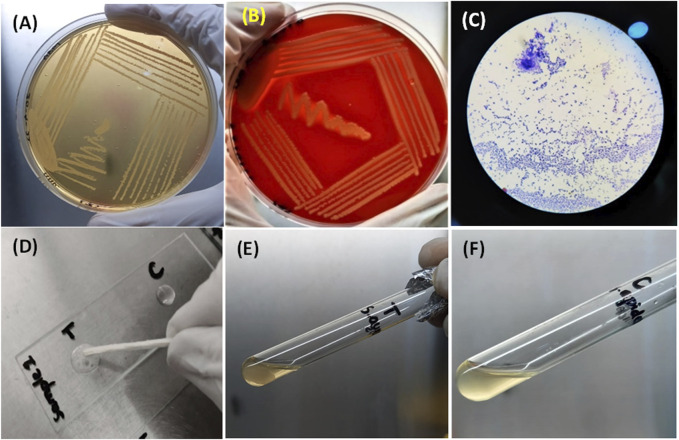
**(A)** Growth of *S. aureus* on MSA showing mannitol fermentation by turning the media yellow. **(B)** Beta-hemolytic colonies of *S. aureus* on blood indicated by a clear zone of hemolysis. **(C)** *S. aur*eus as Gram-positive (purple color) cocci under the microscope. **(D)** Bubble production by the catalase-positive *S. aureus* (left) and no bubble production in the control (right) **(E)** and **(F)** clot formation by *S. aureus* and no clot formation by *S. epidermidis* (control).

All samples of *Staphylococcus aureus* produced small yellow colonies with yellow zones on MSA because of the fermentation of mannitol present in the medium, which produces an acidic byproduct that turns the color of phenol red indicator dye of MSA to yellow (Figure 4A). In contrast, on blood agar, it had β-hemolytic colonies surrounded by a clear zone due to complete lysis of red blood cells. The complete lysis occurred because of the production of hemolysins by *S. aureus* (Figure 4B).

#### Biochemical testing for identification (Gram staining, catalase test, and coagulase test)

All samples of *S. aureus* appeared as purple cocci (Gram-positive) occurring as clusters like the bunches of grapes under the compound microscope after Gram staining (Figure 4C). When the catalase test was performed for *S. aureus*, bubble production was observed because of the production of the catalase enzyme, whereas for the control, no bubble production was observed (Figure 4D). A clot was produced in the test tube after a coagulase test for *S. aureus*, indicating the coagulase enzyme production by *S. aureus.* In comparison, no clot formation occurred in the test tube containing *S. epidermidis* (Figures 4E, F).

#### Antibiotic susceptibility testing

**FIGURE 5:**
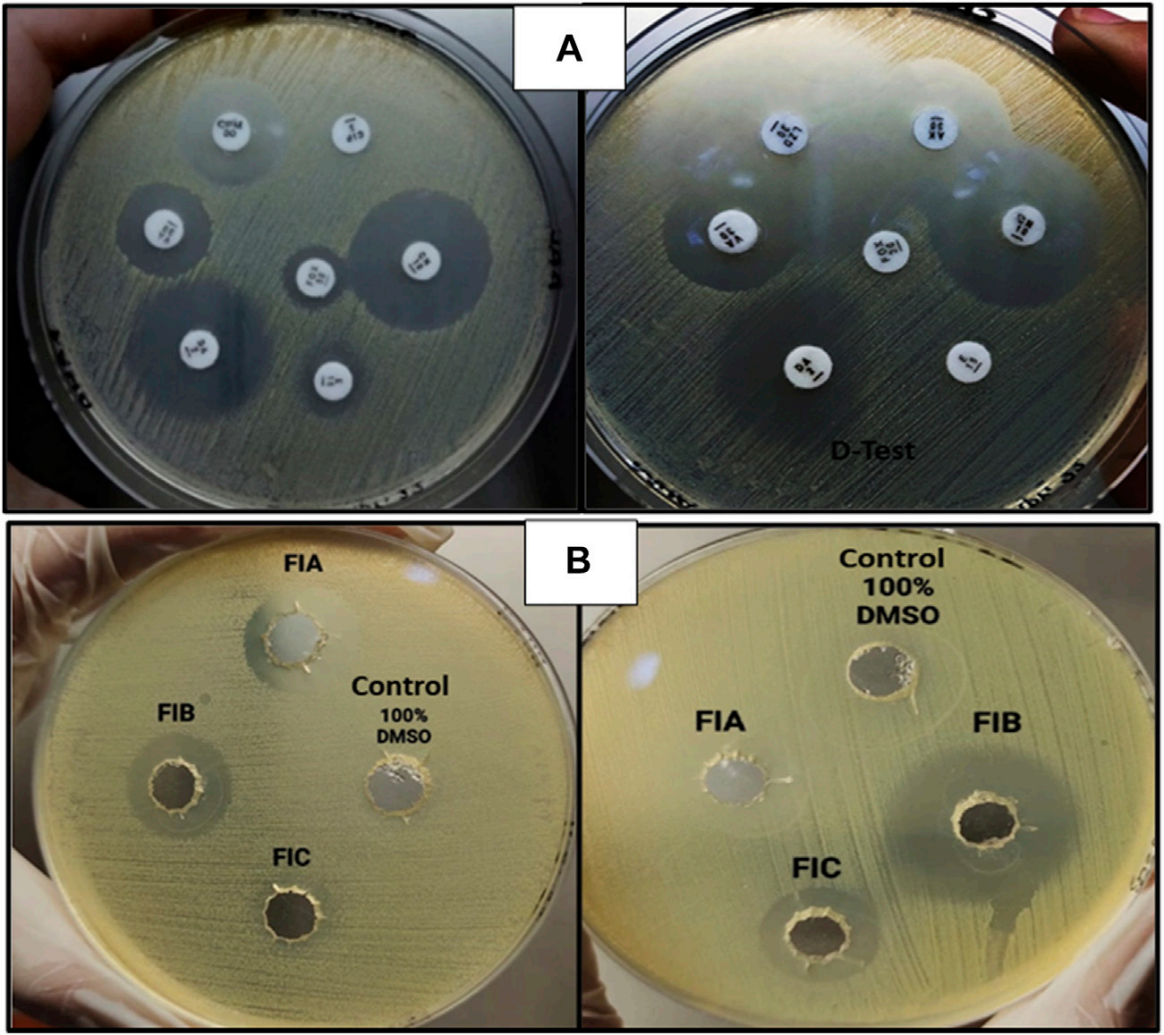
**(A)** AST for MRSA showing clear zones of inhibition (left) and D-test [inducible clindamycin resistance in the presence of erythromycin (right)]. **(B)** Zones of inhibition obtained as a result of agar-well diffusion assay for chiral phthalimides.

The zones obtained for different antibiotics through the Kirby–Bauer disc agar diffusion method, after incubation for 18–24 h, are summarized in Table 4. While measuring the zones of inhibition produced by *S. aureus* (MRSA), the cefoxitin disc needs to be in the resistant range (</ = 21 mm) as it serves as the basis of identification for MRSA. All the samples obtained were resistant to four antibiotics (FOX, P, VA, and CIP) and sensitive to three antibiotics (CN, AK, and LZD), whereas for the antibiotic erythromycin, half of the samples were susceptible, whereas the other half were resistant, which, in turn, resisted clindamycin because of the phenomenon of D-test (Figure 5A). For AST, all the experiments have been performed on all 10 samples of MRSA.

**TABLE 4:** Table for the interpretation of AST.

S. no.	Antibiotic disc	Standard resistant range (CLSI 2022) (mm)	Zone obtained via AST (mm)	Interpretation
1	Cefoxitin (FOX- 30 ug)	≤21	9	RESISTANT (indicating the presence of MRSA)
2	Penicillin (P- 10 ug)	≤28	9	RESISTANT
3	Vancomycin (VA- 30 ug)	≤17	16	RESISTANT
4	Ciprofloxacin (CIP- 5 ug)	≤15	12	RESISTANT
5	Cefepime (CPM- 30 ug)	≤23	10	RESISTANT
5	Gentamicin (CN- 10 ug)	≤12	20	SUSCEPTIBLE
6	Erythromycin (E- 15 ug)	≤13	13	Half of the samples were RESISTANT, whereas half were SUSCEPTIBLE
7	Clindamycin (DA- 2 ug)	≤14	19	SUSCEPTIBLE (but reported as resistant where the spp. was resistant to erythromycin because of the D-test)
8	Amikacin (AK- 30 ug)	≤17	22	SUSCEPTIBLE
9	Linezolid (LZD- 30 ug)	≤20	30	SUSCEPTIBLE

#### Antibacterial activity of chiral phthalimides

The antibacterial activity of chiral phthalimides was evaluated through the agar-well diffusion method. The average zones of inhibition for FIA, FIB, and FIC (conc. 5 mg/ml) were ≤15 mm, ≤21 mm, and ≤12 mm, respectively, and no zone was observed in control (100% DMSO), as shown in Figure 5B. For checking the antibacterial activity of chiral phthalimides, all the experiments were performed in triplicate, and their average is mentioned in the results.

#### MIC through the micro-broth dilution method

The results obtained for MIC are interpreted in Table 5. In the table, the “−“ sign indicates no growth (clear well), and the “+” sign indicates growth (turbid well). The encircled regions in the table indicate the MICs. The minimum inhibitory concentration of a particular compound is one dilution lesser than the dilution at which visible growth has occurred. So, for FIA, FIB, and FIC, the concentrations 0.73 ug, 0.022 ug, and 93 ug per mL of DMSO, respectively, are the MICs. Initially, 10 dilutions were made from the stock solution, but when results were visualized, the MIC for compound 3 (FIC) was obtained only. The last dilution was then diluted 10 times to get the minimum inhibitory concentrations for FIA and FIB. These results were further verified through MBC.

**TABLE 5:** Table for the result interpretation of MIC from the 96-well plate.

Initial concentration (3000 ug/mL)												
Dilutions 1 to 20												
Well dilution	+ve C	1	2	3	4	5	6	7	8	9	10	-ve C
Conc. of compounds in the well (ug/mL)	200ul MF	3000	1500	750	370	180	93	46	23	11	5.8	200ul NB
FIA	+	–	–	–	–	–	–	–	–	–	–	–
FIB	+	–	–	–	–	–	–	–	–	–	–	–
FIC	+	–	–	–	–	–	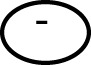 **MIC**	**+**	+	+	+	–
Dilutions 11 to 20												
Well dilution	**+ve C**	**11**	**12**	**13**	**14**	**15**	**16**	**17**	**18**	**19**	**20**	**-ve C**
Concentration of compounds in well (ug/ml)	**200ul MF**	**2.9**	**1.4**	**0.73**	**0.36**	**0.18**	**0.09**	**0.045**	**0.022**	**0.011**	**0.0055**	**200ul NB**
FIA	+	–	–	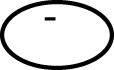 **MIC**	+	+	+	+	**+**	+	+	-
FIB	+	–	–	–	–	–	–	–	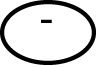 **MIC**	+	+	–
FIC	+	+	+	+	+	+	+	+	+	+	+	-

In the table above, MF indicates McFarland Standard; NB indicates nutrient broth; MIC, Minimum Inhibitory Concentration. The “–“ sign indicates no growth (clear well), and the “+” sign indicates growth. Number range 1–20, indicates the wells of the 96-well plate. Values in (ul), indicates the diluted compound in each well.

#### Determining the minimum bactericidal concentration

The results for MBC on the overnight incubated MHA and blood agar are shown in Supplementary Figure S1, confirming the MBC. The specific concentrations of the chiral phthalimide compounds FIA, FIB, and FIC for MIC and MBC were 0.73 ug, 0.022 ug, and 93 ug/mL of DMSO, respectively. It can be interpreted from the above results that the chiral phthalimides used in this project were bactericidal, killed MRSA, and yielded the same values for MIC and MBC. Furthermore, the results indicate that FIA has the most potent antibacterial activity among all the three chiral phthalimides used.

## Discussion

Antibiotic resistance has been declared a “Global Public Health Concern” by the Centers for Disease Control and Prevention (CDC), WHO, the World Economic Forum, and the Infectious Diseases Society of America. In the past 2 decades, the acquired MDR infections have increased because of the production of beta-lactamase enzymes (including extended-spectrum β-lactamases (ESBLs), carbapenemases, and metallo-β-lactamases), which leads to 3rd-generation cephalosporin and carbapenem resistance (Aslam et al., 2018; Vestergaard et al., 2019). Currently, a group of MDR pathogens collectively known as “ESKAPE” bacteria also includes MRSA, which is considered the most important pathogen due to its resistance, mortality, and clinical importance. So, being a member of a highly pathogenic group of microorganisms, *S. aureus* was selected for this project (Senobar Tahaei et al., 2021). Considering the resistance story of *S. aureus*, it rapidly became MDR over several decades, starting with the production of penicillinase and penicillin resistance early in the 1940s, followed by the macrolide and tetracycline resistance in the 1950s and, finally, the emergence of MRSA (methicillin resistance) in the early 1960s (Morehead and Scarbrough, 2018), showing resistance to β-lactam antibiotics such as methicillin, oxacillin, nafcillin, cloxacillin, and dicloxacillin. This increased resistance rate was attributed to the presence of a “mecA”-resistant gene encoding a “penicillin-binding protein 2a” (PBP2a), which made the MRSA strains more resistant to methicillin (Liu et al., 2016). This is because the active site of PBP2a contains the active site serine (Ser403) located at the N-terminus which lies deep in the pocket and is inaccessible to beta-lactam antibiotics. This closed active site indicates the resistance offered by PBP2a (Bien et al., 2011). Although this multidrug resistance was developed subsequently, it has become a worldwide issue; therefore, the CDC has labeled it a severe threat to the healthcare system (Morehead and Scarbrough, 2018; Senobar Tahaei et al., 2021). Hence, due to the vital role of PBP2a protein in the resistance of MRSA, it was used as a target protein for checking the anti-MRSA activities of the novel chiral phthalimides in the current project.

Considering these susceptibility reports for MRSA (Fri et al., 2020; Senobar Tahaei et al., 2021) mentioned in the literature, we conducted AST using the CLSI guidelines 2022 in our project, and it was examined that all the MRSA isolates showed complete resistance to the antibiotics: cefoxitin (30 ug), penicillin (10 ug), ciprofloxacin (5 ug), vancomycin (30 ug), and cefepime (30 ug), and the highest level of susceptibility to the antibiotics was shown by gentamicin (10 ug), amikacin (30 ug), and linezolid (30 ug), whereas some isolates showed variable degree of resistance to two of the antibiotics, erythromycin (15 ug) and clindamycin (2 ug), because of the phenomenon of the D-test. The results of AST obtained followed the previous studies and indicated a high threat. Therefore, current-day researchers need to use alternative treatment options for microbial resistance. In this regard, an interdisciplinary approach to dealing with the resistance issue is gaining interest in the scientific community. So, our project used methods such as bioinformatics, synthetic chemistry, and microbiology. The emphasis on an interdisciplinary approach, involving bioinformatics for compound selection, synthetic chemistry for compounds synthesis and characterization, and microbiology for their antibacterial actions, ensures a comprehensive investigation. This collaboration increases the study’s depth and validity by highlighting the distinct contributions of each discipline to the overall findings. This research project was focused on using the novel compound “chiral phthalimides” by checking its antibacterial activities under *in silico* and *in vitro* environments against the PBP2a protein of MRSA. This will provide safe preventive and treatment approaches against *S. aureus* infections, contributing to the efficient management of infections and reducing the spread of antimicrobial resistance. The *in silico* part (molecular docking) strengthens the importance of this research project, which was carried out before reporting the *in vitro* activity of chiral phthalimides. The chiral phthalimides were initially docked against the PBP2a protein of MRSA to report their *in silico* antibacterial activity. For this purpose, online computational databases such as NCBI and PubChem were used for retrieving the sequences of the target protein and the compounds, respectively. Once the active sites of PBP2a protein were determined using CASTp, docking of all the three chiral phthalimides was performed using PatchDock. Results generated through molecular docking reported that these compounds gave the best results by interacting with the active site of PBP2a through hydrogen bonds. Compound 1 (FIA) interacted with the amino acid ARG-151, compound 2 (FIB) interacted with the amino acid THR 216, and compound 3 (FIC) interacted with the amino acid THR 216 within the active site of PBP2a through hydrogen bonds. FIB also interacted with PBP2a through a 2nd hydrogen bond (LYS 218) which resides outside the active pocket of the target protein. A similar molecular coupling study was conducted in 2019 in the active pocket of the PBP2a protein of MRSA with the monomeric units of “Eudragit E-100 chloride” (EuCl) and “the sodium salt of poly(maleic acid-alt-octadecene)” (PAM18Na) polymers in both neutral and ionized forms as well as with antibiotic ampicillin. Results suggested that the monomeric forms of both the EuCl and PAM18Na polymers, as well as ampicillin antibiotic, showed interactions of different types and intensities [i.e., hydrophobic interactions (HIs), hydrogen bonds (HBs), and electrostatic interactions (EIs)] with the amino acids in the active site of PBP2a which, in turn, suggested antibacterial activities against the resistant strains of *S. aureus* (Liscano et al., 2021). Another docking study was conducted in 2016 for the synergistic effects of metronidazole–triazole hybrids with oxacillin against PBP2a of MRSA. The results of the docking studies and docking confirmations for these MTZ–triazole hybrids gave the best scores in the active pocket of PBP2a by interaction through hydrogen bonds as well as π–π interactions in the predicted binding pockets (Negi et al., 2016).

After getting the results for molecular docking, the *in vitro* antibacterial activity of the selected chiral phthalimides was checked against MRSA using standard protocols. Different zones of inhibition were obtained for FIA, FIB, and FIC, indicating positive outcomes for their antibacterial activity. A related study carried out in Egypt by Lamie et al. has reported the antibacterial and antifungal activities of the novel phthalimide derivative against the fungus (*C. albicans*), as well as Gram-positive and Gram-negative bacteria, i.e., *Bacillus subtilis* and *P. aeruginosa*, respectively, by performing the agar-well diffusion assay. Similarly, those compounds were characterized to be non-cytotoxic after completing the cytotoxicity assay in cancer and normal human cell lines (Lamie et al., 2015). From this study, it may be assumed that our synthesized chiral phthalimides (FIA, FIB, and FIC) may be non-toxic in normal therapeutic concentration ranges. Still, the cytotoxicity of these chiral phthalimides is recommended for further confirmation.

To further evaluate the antibacterial activity of these compounds, we performed the micro-broth dilution method (96-well plate method) and visual plate assay to identify the minimum inhibitory and minimum bactericidal concentrations of these chiral phthalimides. All the compounds showed excellent anti-MRSA activities, with FIB being the strongest anti-staphylococcal agent having an MIC of 0.022 ug (probably because of the structural differences in FIB and two covalent bonds formed with the PBP2a of MRSA in contrast to only one covalent bond in case of FIA and FIC) compared to FIA and FIC, which have MICs of 0.73 ug and 93 ug/mL of DMSO, respectively. In our study, the MIC and MBC values obtained for these chiral phthalimides were the same, meaning our compounds were bactericidal at the lowest concentration. This strengthens our research because bactericidal compounds are usually preferred over bacteriostatic compounds due to the complete elimination of the pathogenic agent from a particular site rather than just inhibiting its growth. A related study conducted in Bangladesh by Nayab et al. (2015) reported the antibacterial, antioxidant, and DNA-binding activities of the novel phthalimide derivatives, where significant antibacterial activity was shown by the novel N-substituted phthalimides against *E. coli* and *S. mutans*. Similarly, the chiral phthalimides used in our project along with some additional derivatives, i.e., FIB and FIC, as well as FIF: 2-(1,3-dioxoisoindolin-2yl)-3 mercaptopropionic acid, FIH: 2-(1,3-dioxoisoindolin-2yl)-3 (4-hydroxyphenyl prophanic acid, and FII: 3-(1,3-dioxoisoindolin-2yl) prophanic acid, have been reported to have good antibacterial activities against the MDR hypervirulent strain of *Klebsiella pneumonia* (hvKp) when the agar-well diffusion method and micro-broth dilution assay were performed in a similar study conducted at IPDM, Khyber Medical University. Similarly, these chiral phthalimides were reported to have no cytotoxic effects (non-cytotoxic) when their cytotoxicity assay was performed against human red blood cells (RBCs) (Khan et al., 2022).

As the results obtained during our research project align with the findings of similar studies conducted in the past, it confirms that, yes, the chiral phthalimides used against MRSA possess good antibacterial activities and could be a possible treatment option for the drug-resistant staphylococcal infections in future. Moreover, to our understanding, this is a novel study because no such studies have been explicitly conducted against *S. aureus* previously. These findings provide useful insights within the defined area of the investigation, despite the fact that the current study has a limited sample size of ten MRSA due to resource limitations. The findings of this study add to the existing body of information and have the potential to serve as a basis for additional research.

In conclusion, the chiral phthalimides exhibited antibacterial activities against multidrug-resistant MRSA and have the potential to be considered as alternative chemotherapeutics to treat the infections caused by MRSA after the confirmation of their cytotoxic and pharmacokinetic studies. The observed activities highlight their importance in the fight against antibiotic-resistant strains. Recognizing the need for a comprehensive assessment, future research will concentrate on cytotoxicity and pharmacokinetics. Confirming these aspects will help us better understand the safety and efficacy profiles of chiral phthalimides, positioning them as potential candidates for treating MRSA infections. This study lays the groundwork for further investigation into the full therapeutic potential of these compounds, providing a potential solution to the problems caused by MRSA infections.

## Data Availability

The datasets presented in this study can be found in online repositories. The names of the repository/repositories and accession number(s) can be found in the article/Supplementary Material.
