# Supplementary material for: Chiral phthalimides against penicillin-binding protein 2a of methicillin-resistant Staphylococcus aureus: molecular docking and in vitro analysis
Source: Front Pharmacol. 2024 Feb 28;15:1293458. doi: 10.3389/fphar.2024.1293458 (PMC10932970; doi:10.3389/fphar.2024.1293458)
Supplement: Supplementary file 1 [file Table1.DOCX]

**Title: Chiral Phthalimides against Penicillin-binding protein2a of Methicillin-resistant *Staphylococcus aureus*: Molecular Docking and *In vitro* Analysis**

Aamina Azam Khan^1^, Momin Khan^2^*, Sher Wali Khan^3^, Nayyer Siddique^4^, Rimsha Abid^1^, Shandana Zulfiqar^1^, Sidra Rahman^5^, Muhammad Ali^5^*

^1^*Institute of Pathology and Diagnostic Medicine, Khyber Medical University (KMU), Peshawar, Pakistan.*

^2^*Department of Microbiology, Khyber Medical University (KMU), Peshawar, Khyber Pakhtunkhwa, Pakistan.*

^3^*Department of Chemistry, Shaheed Benazir Bhutto University (SBBU), Sheringal, Khyber Pakhtunkhwa, Pakistan.*

^4^*Institute of Basic Medical Science, Khyber Medical University (KMU), Peshawar, Khyber Pakhtunkhwa, Pakistan.*

*^5^Department of Biotechnology, Quaid-i-Azam University Islamabad, Pakistan-45320*

-------------------------------------------------

***CORRESPONDING AUTHORS**

*Momin Khan Email address:* [*mominkhan.ibms@kmu.edu.pk*](mailto:mominkhan.ibms@kmu.edu.pk)

*Muhammad Ali* *Email address:* [*muhammad.ali@qau.edu.pk*](mailto:muhammad.ali@qau.edu.pk)

**Supplementary material**

**Table S1.** Amino acids present in the binding pockets of PBP2a were determined through CASTp

| **Binding Pockets of PBP2a** | | | | | | | | |
| --- | --- | --- | --- | --- | --- | --- | --- | --- |
| 68:  Lysine | 143:  Histidine | 151:  Arginine | 203:  Glutamate | 213:  Proline | 241:  Arginine | 274:  Aspartate | 295:  Aspartate | 374:  Glycine |
| 71:  Asparagine | 144:  Isoleucine | 165:  Threonine | 204:  Lysine | 214:  Leucine | 256:  Valine | 275:  Alanine | 296:  Glycine |  |
| 72:  Serine | 145:  Glutamate | 170:  Glutamate | 205:  Tryptophan | 215:  Lysine | 257:  Glycine | 276:  Alanine | 297:  Tyrosine |  |
| 73:  Leucine | 146:  Lysine | 196:  Tyrosine | 207:  Glutamine | 216:  Threonine | 258:  Proline | 277:  Valine | 307:  Asparagine |  |
| 74:  Glycine | 148:  Lysine | 199:  Glutamine | 209:  Aspartate | 238:  Threonine | 271:  Glycine | 292:  Glutamine | 316:  Glycine |  |
| 104:  Asparagine | 149:  Serine | 200:  Lysine | 210:  Threonine | 239:  Glutamate | 272:  Tyrosine | 293:  Histidine | 372:  Methionine |  |
| 105:  Tyrosine | 150:  Glutamate | 202:  Aspartate | 212:  Valine | 240:  Serine | 273:  Lysine | 294:  Glutamate | 373:  Tyrosine |  |


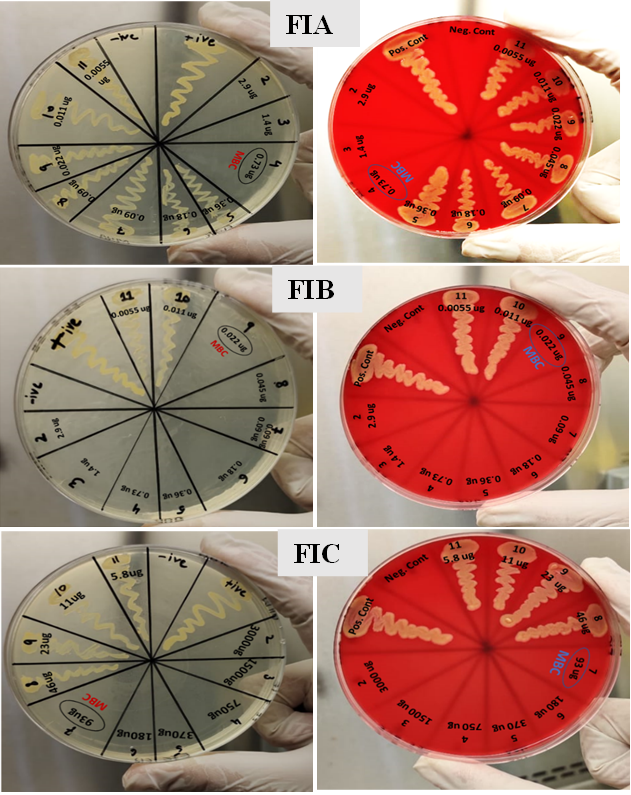


**FIGURE S1.** MBC for FIA, FIB and FIC on MHA (left) and blood agar plates (right)
